# Does physical activity buffer insomnia due to back and neck pain?

**DOI:** 10.1371/journal.pone.0184288

**Published:** 2017-09-20

**Authors:** Iben Axén, Lydia Kwak, Jan Hagberg, Irene Jensen

**Affiliations:** Karolinska Institutet, Institute of Environmental Medicine, Unit of Intervention and Implementation for Worker Health, Nobels v 13, Stockholm, Sweden; University of Rome Tor Vergata, ITALY

## Abstract

**Introduction:**

Musculoskeletal pain is highly prevalent and a burden to society, recurrent and persistent low back pain (LBP) and neck pain (NP) being the most common conditions. They are associated with other poor health outcomes such as sleep problems.

Physical activity (PA) prevents LBP and NP, but the direct effect on sleep is unclear. This study explored the effect of pain on insomnia, and examined if adherence to moderate-to-high intensity levels of PA influenced this relationship.

**Methods:**

In this prospective observational study, 1821 workers were followed over 3 years. Data included self-rated measures of LBP and NP, insomnia and level and amount of PA. Pain variables were used in a “risk profile” for future sick-listing, insomnia was categorized into those with and without such problems, and adherence to PA was defined as reporting moderate-to-high levels in two consecutive years.

In Poisson regression models, individuals with pain risk profiles were analysed according to PA adherence for the outcome insomnia. Repeated measurements allowed control for prior pain.

**Results:**

In this mainly male working population, individuals with a risk profile for LBP and NP had a significant increased risk (RR = 1.5) of developing insomnia one year later when not adhering to moderate-to-high levels of PA. Among those not reporting prior pain, the risk was even larger (RR = 2.5).

Generalizability may be restricted to relatively healthy males. The individuals who reported a pain risk profile two consecutive years did not get the buffer effect from adhering to moderate-to-high levels of PA in terms of developing insomnia.

## Introduction

Musculoskeletal pain is a global health burden, the major cause of years lived with disability [1]. This is due to a high prevalence and a high level of recurrence and persistence [2, 3]. On the societal level, the burden is economic and relates to direct costs associated with health care, as well as indirect costs for disability pensions [4, 5]. From the employer level, musculoskeletal pain leads to decreased work productivity, sickness presentism and absenteeism, all are costly for the company [6].

For the individual, the numerous effects are well documented. Aside from decreased work ability and possibly sick leave, pain also affects health in general [7]. People with persistent pain report poor general health [8], comorbidity [9]and poor sleep [10]. Thus, these individuals are caught in a cycle of negative health outcomes, one problem adding insult to the other [9]. As mentioned, musculoskeletal pain tends to be persistent, which means that the individual needs to develop strategies to manage their pain [11]. Preferably, preventive measures should be implemented to minimize recurrence and the level of interference with everyday life.

Sleep has consistently been found to be affected among those suffering from musculoskeletal pain. Reports of decreased sleep quality [12] and disruption of sleep [12] concern patients with low back pain (LBP), neck pain (NP), headache and jaw pain. In a previous prospective study of self–reported bothersome LBP and decreased sleep quality, pain predicted disturbed sleep the following week [13]. In a study with an 11 year follow up, headache and musculoskeletal pain was found to predict insomnia [14]. Indeed, musculoskeletal pain and insomnia seem to be co-existing [15]. A recent critical review suggested that the relationship between pain and sleep may be very complex involving neurotransmitters, psychological affect and sociodemographic factors[16].

The only factor that has been consistently shown to prevent LBP and NP, is physical activity (PA) [17]. However, PA is typically a “perishable” entity [18, 19]; it needs to be performed on a regular basis for the beneficial effects to take place. In today’s society, people are becoming more and more sedentary, and much of the “normal” everyday PA is lost [20]. Further, a recent study found that one of the consequences of LBP is physical inactivity [21], adding further insult to the pain problem. However, a systematic review concluded that the direct effect of exercise on sleep is unclear [22].

In this study, the prospective relationship between LBP, NP and insomnia was explored, and the effect of PA on this relationship. It was a secondary analysis of existing data from a workplace inventory concerning workers’ health, lifestyle and productivity. The organisations that supplied data were large Swedish industrial companies, and data existed from 1821 employees measured at three time points, in 2000, 2001–2 and 2003. The aim of the study was to explore the relationship between LBP/NP and insomnia, and to investigate the influence of adhering to moderate-to-high levels of PA on this relationship.

Specific hypotheses were

1. Future insomnia is predicted by LBP and NP.
2. PA influences this relationship; in such a way that individuals with LBP/NP who adhere to moderate-to-high levels of PA over time experience less insomnia compared to individuals who do not adhere.

## Materials and method

### Design

The data were collected as part of a work place intervention study (the AHA study) in 2000 (T1), 2001–2 (T2) and 2003 (T3) in private companies in Sweden. The methodology has been described in detail elsewhere [23]. In short, the inventory consisted of a comprehensive validated individual questionnaire about health and work conditions which was sent by mail to all employees, regardless of their work–status (working full–or part–time, being on parental leave or sick leave). The survey resulted in a personal profile for each participant as well as company profiles, both of which highlighted areas with room for improvement concerning health (individual level) and work–environment (organisational level) [24]. The individuals with a risk profile concerning health issues were recommended to seek advice from their occupational health service, and the companies were directed towards improvements in the work environment and leadership if productivity could be improved. After a period of 18 months, the questionnaire was repeated, the improvement was evaluated, and another round of measures towards individual health and productivity was started. After an additional 18 months, the process was repeated a third time. In the original study, the AHA method was shown to improve workers’ health and the companies’ productivity [23, 25].

### Variables

Demographic and health variables were collected as part of the AHA questionnaire, such as insomnia, the presence and consequences of LBP and NP and patterns of PA.

Concerning sleep, three questions concerning insomnia from the Karolinska Institutet Sleep Questionnaire [26] were asked; about difficulties in falling asleep, in waking up and about having difficulty going back to sleep after waking up. Insomnia is defined by the National Sleep Foundation as “difficulty falling asleep, difficulty staying asleep, waking up too early, and/or having sleep that is not refreshing” [27]. Based on previous research [23], this variable was summarized into three categories: sleep interference on all three dimensions (“severe insomnia”) to no sleep interference on all three dimensions (“no insomnia”) with an intermediate category with “moderate insomnia”.

Questions concerning musculoskeletal pain were based on von Korff et al, [28], and asked about present and past year LBP and NP and present and past year sickness absence in relation to such pain. From these questions, it was possible to create a “high risk pain profile”–a worker with several past episodes and present pain and sick leave more than once the previous year or ongoing for more than 2 months. In a previous study, these individuals were found to be at risk for future sick–leave due to LBP and NP [29]. Likewise, a “no risk pain profile” worker would report no past or recurrent LBP/NP and no long period or repeated sick leave. An intermediate category consisted of individuals with some, but not all, risk factors present [23].

Questions concerning PA were from the modified Swedish “LIV 90” questionnaire [30] and asked about the frequency of performing more than 30 minutes of high, medium and low intensity exercise. From these questions three PA groups were created; those reporting moderate or high intensity exercise more than twice a week or both high/medium intensity exercise once a week were the “high PA” group, those reporting high or medium at a lower frequency were “medium PA”, and those reporting high or medium intensity PA irregularly or never, were termed “low PA”.

Self–Rated Health was measured with a single item question from the Short Form–Questionnaire: “How would you rate your general health?” with five answer options ranging from excellent to poor [31].

### Data analysis

By examining the dependant variable “insomnia”, it was noted that very few individuals reported severe insomnia. As a result, the variable was dichotomized into those without vs those with insomnia (which included the categories moderate and severe). To exclude individuals with persistent insomnia, we chose to examine only those individuals without any “previous” insomnia, at baseline (T1 and T2) (Model 1). This amounted to 436 individuals. As a next step, this group was dichotomized into those with a pain risk profile also at T1 (Model 2, n = 347) and those that did not (Model 3, n = 85).

The independent variable LBP/NP risk profile classified very few subjects in the “high” risk category, and this group was collapsed with the “medium” risk category into a dichotomized index with risk/no risk of LBP/NP.

The independent variable PA was introduced in the model as “PA adherence”. This is a conceptualization of maintaining PA levels over time. Thus, if individuals started reporting moderate or high PA levels at T2 and maintained one of these levels at T3 (individuals with high PA levels could decrease their PA to moderate, but individuals with moderate PA levels needed to maintain this or increase to high PA levels) and individuals who started reporting low PA levels at T2 but increased to moderate or high PA levels at T3 were categorized as having “PA adherence”. All individuals who reported low levels of PA at T3 were classified as “PA non-adherence”.

In regression models, individuals with LBP and NP risk profiles at T2 were analysed according to adherence to their levels of PA (between T2 and T3) for insomnia at T3. Due to the repeated measurements, it was possible to control for prior pain (as reported at T1). Poisson regression was chosen to get estimates of risk ratios. Self–Rated Health was introduced as a covariate in the models.

We explored the following models (Tables 1–3):

**Table 1:** Model 1: Examining the effect of PA adherence on subjects with LBP/NP risk profiles at T2 on the risk of developing insomnia at T3, regardless of previous pain at T1 (n = 436).

TIME	T1	T2		T3
**RISK OF LBP & NP**	All subjects, both yes/no	Yes	PA adherence T2–T3	Insomnia
**INSOMNIA**	No	No		

**Table 2:** Model 2: Examining the effect of PA adherence on subjects with LBP/NP risk profiles that also reported pain at T1, on the risk of developing insomnia (n = 347).

TIME	T1	T2		T3
**RISK OF LBP & NP**	Yes	Yes	PA adherence T2–T3	Insomnia
**INSOMNIA**	No	No		

**Table 3:** Model 3: Examining the effect of PA adherence on subjects with LBP/NP risk profiles that did not report pain at T1, on the risk of developing insomnia (n = 85).

TIME	T1	T2		T3
**RISK OF LBP & NP**	No	Yes	PA adherence T2–T3	Insomnia
**INSOMNIA**	No	No		

Ethical approval was granted by Karolinska Institutet: 00–012

## Results

Demographic variables of 1821 workers who participated at all three measurements were available for secondary analysis. Of these, subgroups of individuals who did not report insomnia at the initial measurements in T1 and T2 and who reported a LBP and NP risk profile at T2 formed the study sample in Model 1. This subsample was further divided according to pain at T1 into Model 2 (pain risk profile at T1) and Model 3 (no pain risk profile at T1).

The profiles of these individuals are described in Table 4. As mentioned, these were workers from private, mainly industrial companies, which is reflected in the high proportion of males (around 90%) and also in the high proportion of subjects with primary and secondary schooling (around 90%). A majority worked full time (40 hours a week in Sweden), but a fair proportion (over 30%) worked more than that. A relatively high proportion reported moderate-to- high PA levels in all the subsamples. About two–thirds of the cohort adhered to moderate-to-high PA levels during the course of the study, from T2 to T3. Self-Rated Health was higher in the last subsample, three quarters of individuals reported excellent/very good health, compared to half the original sample. Apart from this difference, the subsamples were similar.

**Table 4:** Baseline information about the source population and the selected subgroups.

	Full study n = 1821	Model 1 n = 451	Model 2 n = 361	Model 3 n = 86
**Age** mean (SD)	43 (10.12)	44 (10.08)	43 (9.91)	44 (10.86)
**Males**	87.5%	90.2%	89.2%	94.2%
**Education**				
University-tertiary Secondary Primary	9.5% 58.1% 32.4%	8.7% 58.2% 33.1%	9.0% 59.7% 31.4%	8.1% 53.5% 38.4%
**Children at home**				
0 1–2 3 or more	31.1% 55.7% 14.2%	33.0% 52.8% 14.2%	30.6% 54.5% 14.9%	43.1% 47.1% 9.8%
**Work hours/week**				
1–20 21–30 31–40 41 +	2.3% 2.5% 63.8% 31.4%	1.1% 1.4% 63.2% 36.8%	1.4% 1.0% 64.6% 33.1%	0% 3.5% 57.0% 39.5%
**BMI**				
< 24.9- normal 25–29.9-overweight 30+ obese	39.3% 47.4% 13.3%	39.6% 47.0% 13.4%	40.8% 48.1% 11.1%	34.9% 42.4% 22.4%
**LBP/NP risk profile**	n = 1792			
High + Moderate No risk	1249–69% 543–31%			
**PA levels**	T1	T2	T2	T2
High Moderate Low	827–45% 190–10% 794–44%	244–55% 47–11% 153–34%	191–54% 40–11% 124–35%	51–60% 7–8% 27–32%
**PA adherence between T2 and T3**				
Non-adherence Adherence		164 (38%) 272 (62%)	125 (36%) 222 (64%)	36 (42%) 49 (58%)
**Insomnia**				
Severe Moderate No	147–8% 657–36% 989–55%			
**Self–Rated Health**		T2	T2	T2
Excellent Very Good Good All right Poor	236–13% 607–33% 705–39% 234–13% 37–2%	67–15% 187–42% 167–37% 29–6% 1–0.3%	38–11% 150–42% 145–40% 27–7% 1–0.3%	28–33% 37–43% 20–23% 1–1%

The modified Poisson regression analysis (Model1) showed that individuals with LBP/NP risk profiles at T2 independently of back problems or not at T1 and without insomnia both at T1 and T2, significantly increased the risk of insomnia at T3 (RR = 1.5) if they reported poor adherence to moderate-to-high levels of PA (Table 5).

**Table 5:** The risk ratios of three models investigating the risk of reporting future insomnia among individuals with a LBP/NP risk profile. * = statistical significance.

	PA adherence T2–T3	RR (CI) of Reporting insomnia at T3	p–value
Model 1 (n = 436)	Non-adherence	1.528 (1.01–2.32)	0.046*
	Adherence	1	
Model 2 (n = 347)	Non-adherence	1.332 (0.82–2.16)	0.243
	Adherence	1	
Model 3 (n = 85)	Non-adherence	2.496 (1.02–6.12)	0.046*
	Adherence	1	

However, for individuals with LBP/NP risk profiles repeatedly over a longer time period (18 months) i.e. both at T1 and T2 (Model 2), adherence to moderate-to-high PA levels had no impact on reporting insomnia at T3.

Finally, among individuals with LBP/NP risk profile only at T2 (i.e. reported no pain at T1), the risk of insomnia was significantly higher (RR = 2.5) among those with poor adherence to moderate-to-high PA levels. The risk ratios of these models are shown in Table 5.

When introducing Self–Rated Health as a covariate in the model, the estimates were slightly smaller, and PA in Models 1 and 3 were no longer significant (results not shown). No interactions with the independent variables were found. Likely, Self–Rated Health is a construct that is mirrored partly by pain and PA. Therefore, it did not make sense to keep it in the model.

## Discussion

Among a mainly male working population followed over 3 years, those with a LBP/NP risk profile had a significantly increased risk (RR = 1.5) of developing insomnia one year later when not adhering to moderate-to-high PA levels. Among those who had a LBP/NP risk profile two years in a row, the risk of insomnia was not significantly affected by PA adherence. However, among those without a LBP/NP risk profile at T1, i.e. only at T2, the risk of insomnia was significantly higher (RR = 2.5) among those not adhering to moderate- to-high PA levels compared to those who were physically active. Thus, PA seems to buffer insomnia among those with a LBP/NP risk profile, provided this profile does not persist two years in a row.

The main strength of the study was the sample size of workers followed for three years. This enabled subgroup analysis by adherence to PA guideline, and also made the exploration of temporality possible. Thus, the difference between workers with LBP/NP risk profiles at both measuring points (T1 and T2) and those reporting it only later in the study (T2) could be highlighted.

The variables in this study are based on validated questionnaires and resulting indexes for pain, PA and sleep. It could be argued that the measurements on which this study are based were “coincidental moments in time”, reflecting the intermittent nature of LBP and NP. The time points may, however, fail to capture individuals with persistent of pain. This is relevant considering the finding that in subjects with LBP/NP risk profiles at T1 and T2, adherence to the moderate-to-high levels of PA did not influence the risk of insomnia. It may be that these individuals were in fact suffering from “chronic” LBP/NP, but the measurements may just reflect arbitrary moments in time and may not be adequate to explore temporality. In a previous study from Sweden [32], insomnia was found to only partly explain the development of chronic pain, thus the “direction of risk” may be less important as pain become more widespread and persistent. A systematic review examined the effect of exercise on insomnia in middle-aged women [33] and found that PA did not affect insomnia among these individuals. Possibly, there are also differences between the sexes (our sample was mainly male) concerning the ability of PA to buffer insomnia, Another recent systematic review examined non-pharmacological interventions for insomnia in individuals with persistent pain, but did not include interventions of exercise [34].

The data stem from self–rated measures only. Self–reported PA is known to be over–estimated, and objective measures are recommended. Moreover, little is known regarding the validity of self-reported PA measures among populations with pain. The PA questions in this study were constructed before the World Health Organisation’s recommendations of 150 minutes of activity every week were made [35], and the levels reported herein are therefore difficult to assess in term of adequate PA levels. Adherence to levels of PA was a conceptualization made to illustrate the long–term effects of health enhancing PA as opposed to examining PA levels only in a cross–sectional manner. However, the underlying assumption of this measure was that the variable PA was constant if the same levels were reported at two consecutive time points, which could be an overestimation of the true adherence to PA levels over time. In this case, objective measures would have been preferable. Objective measures can measure total PA over a prolonged period of time, including PA performed in different domains, such as work, leisure time and transport. This would give the opportunity to assess a possible differential impact of PA performed during these different domains.

By selecting only those without insomnia at T1 and T2, we probably selected a slightly healthier subgroup of the source population; indeed 76% rated their health as very good or excellent as opposed to 46% in the full population. These subjects were relatively adherent [36] to moderate-to-high levels of PA, which is a further indication of a healthy worker effect. In a recent American study, adherence to recommended PA levels were found among the slightly older and healthy [37], which is in agreement with these results.

Interestingly, Self-Rated Health changed the estimates just enough to make them non-significant. It may be due to the fact that both pain, sleep and PA are factors contributing to the perceived health of an individual, and controlling for health in our models dilutes the effect of each variable.

In conclusion, PA adherence has the ability to buffer the effect of LBP/NP pain on future insomnia. However, in individuals reporting LBP/NP risk profiles over two consecutive years, PA does not significantly alter this risk.

## References

[1] Vos T, Flaxman AD, Naghavi M, Lozano R, Michaud C, Ezzati M, et al Years lived with disability (YLDs) for 1160 sequelae of 289 diseases and injuries 1990–2010: a systematic analysis for the Global Burden of Disease Study 2010. Lancet. 2012;380(9859):2163–96. Epub 2012/12/19. doi: 10.1016/S0140-6736(12)61729-2 . 23245607 10.1016/S0140-6736(12)61729-2 PMC6350784

[2] Axen I, Leboeuf-Yde C. Trajectories of low back pain. Best practice & research. 2013;27(5):601–12. Epub 2013/12/10. doi: 10.1016/j.berh.2013.10.004 . 24315142 10.1016/j.berh.2013.10.004

[3] Leboeuf-Yde C, Fejer R, Nielsen J, Kyvik KO, Hartvigsen J. Consequences of spinal pain: do age and gender matter? A Danish cross-sectional population-based study of 34,902 individuals 20–71 years of age. BMC Musculoskelet Disord. 2011;12:39 Epub 2011/02/09. doi: 10.1186/1471-2474-12-39 ; PubMed Central PMCID: PMC3041727. 21299908 10.1186/1471-2474-12-39 PMC3041727

[4] Ekman M, Johnell O, Lidgren L. The economic cost of low back pain in Sweden in 2001. Acta Orthop. 2005;76(2):275–84. . 16097556 10.1080/00016470510030698

[5] March L, Smith EU, Hoy DG, Cross MJ, Sanchez-Riera L, Blyth F, et al Burden of disability due to musculoskeletal (MSK) disorders. Best practice & research. 2014;28(3):353–66. Epub 2014/12/08. doi: 10.1016/j.berh.2014.08.002 . 25481420 10.1016/j.berh.2014.08.002

[6] Bevan S. Economic impact of musculoskeletal disorders (MSDs) on work in Europe. Best practice & research. 2015;29(3):356–73. Epub 2015/11/28. doi: 10.1016/j.berh.2015.08.002 . 26612235 10.1016/j.berh.2015.08.002

[7] Pueyo MJ, Suris X, Larrosa M, Auleda J, Mompart A, Brugulat P, et al [Importance of chronic musculoskeletal problems in the population of Catalonia (Spain): prevalence and effect on self-perceived health, activity restriction and use of health services]. Gac Sanit. 2012;26(1):30–6. Epub 2011/07/08. doi: 10.1016/j.gaceta.2011.03.008 . 21733600 10.1016/j.gaceta.2011.03.008

[8] Kovacs FM, Abraira V, Zamora J, Fernandez C. The transition from acute to subacute and chronic low back pain: a study based on determinants of quality of life and prediction of chronic disability. Spine. 2005;30(15):1786–92. . 16094282 10.1097/01.brs.0000172159.47152.dc

[9] Schur EA, Afari N, Furberg H, Olarte M, Goldberg J, Sullivan PF, et al Feeling bad in more ways than one: comorbidity patterns of medically unexplained and psychiatric conditions. J Gen Intern Med. 2007;22(6):818–21. Epub 2007/05/16. doi: 10.1007/s11606-007-0140-5 ; PubMed Central PMCID: PMC2219854. 17503107 10.1007/s11606-007-0140-5 PMC2219854

[10] Campbell P, Tang N, McBeth J, Lewis M, Main CJ, Croft PR, et al The role of sleep problems in the development of depression in those with persistent pain: a prospective cohort study. Sleep. 2013;36(11):1693–8. Epub 2013/11/02. doi: 10.5665/sleep.3130 ; PubMed Central PMCID: PMC3792387. 24179303 10.5665/sleep.3130 PMC3792387

[11] Benjaminsson O, Biguet G, Arvidsson I, Nilsson-Wikmar L. Recurrent low back pain: relapse from a patients perspective. J Rehabil Med. 2007;39(8):640–5. Epub 2007/09/27. doi: 10.2340/16501977-0104 . 17896056 10.2340/16501977-0104

[12] van de Water AT, Eadie J, Hurley DA. Investigation of sleep disturbance in chronic low back pain: an age- and gender-matched case-control study over a 7-night period. Man Ther. 2011;16(6):550–6. Epub 2011/06/10. doi: 10.1016/j.math.2011.05.004 . 21652257 10.1016/j.math.2011.05.004

[13] Axen I. Pain-related Sleep Disturbance: A Prospective Study With Repeated Measures. The Clinical journal of pain. 2016;32(3):254–9. Epub 2015/05/15. doi: 10.1097/AJP.0000000000000249 . 25968449 10.1097/AJP.0000000000000249

[14] Odegard SS, Sand T, Engstrom M, Zwart JA, Hagen K. The impact of headache and chronic musculoskeletal complaints on the risk of insomnia: longitudinal data from the Nord-Trondelag health study. J Headache Pain. 2013;14(1):24 Epub 2013/04/10. doi: 10.1186/1129-2377-14-24 ; PubMed Central PMCID: PMC3620476. 23566158 10.1186/1129-2377-14-24 PMC3620476

[15] Baker S, McBeth J, Chew-Graham CA, Wilkie R. Musculoskeletal pain and co-morbid insomnia in adults; a population study of the prevalence and impact on restricted social participation. BMC Fam Pract. 2017;18(1):17 doi: 10.1186/s12875-017-0593-5 ; PubMed Central PMCID: PMCPMC5297165. 28173767 10.1186/s12875-017-0593-5 PMC5297165

[16] Finan PH, Goodin BR, Smith MT. The association of sleep and pain: an update and a path forward. J Pain. 2013;14(12):1539–52. Epub 2013/12/03. doi: 10.1016/j.jpain.2013.08.007 ; PubMed Central PMCID: PMC4046588. 24290442 10.1016/j.jpain.2013.08.007 PMC4046588

[17] Saragiotto BT, Latimer J. Prevention of low back pain (PEDro synthesis). Br J Sports Med. 2016 Epub 2016/04/17. doi: 10.1136/bjsports-2016-096182 . 27084881 10.1136/bjsports-2016-096182

[18] Picavet HS, Wendel-vos GC, Vreeken HL, Schuit AJ, Verschuren WM. How stable are physical activity habits among adults? The Doetinchem Cohort Study. Med Sci Sports Exerc. 2011;43(1):74–9. Epub 2010/05/18. doi: 10.1249/MSS.0b013e3181e57a6a . 20473224 10.1249/MSS.0b013e3181e57a6a

[19] Kubo K, Ikebukuro T, Yata H, Tsunoda N, Kanehisa H. Time course of changes in muscle and tendon properties during strength training and detraining. J Strength Cond Res. 2010;24(2):322–31. Epub 2009/12/10. doi: 10.1519/JSC.0b013e3181c865e2 . 19996769 10.1519/JSC.0b013e3181c865e2

[20] Hagstromer M, Kwak L, Oja P, Sjostrom M. A 6 year longitudinal study of accelerometer-measured physical activity and sedentary time in Swedish adults. J Sci Med Sport. 2015;18(5):553–7. Epub 2014/10/04. doi: 10.1016/j.jsams.2014.07.012 . 25277849 10.1016/j.jsams.2014.07.012

[21] Yang H, Haldeman S. Behavior-related Factors Associated with Low Back Pain in the US Adult Population. Spine (Phila Pa 1976). 2016 Epub 2016/04/30. doi: 10.1097/BRS.0000000000001665 . 27128393 10.1097/BRS.0000000000001665

[22] Driver HS, Taylor SR. Exercise and sleep. Sleep Med Rev. 2000;4(4):387–402. doi: 10.1053/smrv.2000.0110 . 12531177 10.1053/smrv.2000.0110

[23] Bergstrom G, Bjorklund C, Fried I, Lisspers J, Nathell L, Hermansson U, et al A comprehensive workplace intervention and its outcome with regard to lifestyle, health and sick leave: the AHA study. Work. 2008;31(2):167–80. . 18957735

[24] Wannstrom I, Peterson U, Asberg M, Nygren A, Gustavsson JP. Psychometric properties of scales in the General Nordic Questionnaire for Psychological and Social Factors at Work (QPS): confirmatory factor analysis and prediction of certified long-term sickness absence. Scand J Psychol. 2009;50(3):231–44. Epub 2008/11/29. doi: 10.1111/j.1467-9450.2008.00697.x . 19037910 10.1111/j.1467-9450.2008.00697.x

[25] Lohela M, Bjorklund C, Vingard E, Hagberg J, Jensen I. Does a change in psychosocial work factors lead to a change in employee health? Journal of occupational and environmental medicine / American College of Occupational and Environmental Medicine. 2009;51(2):195–203. Epub 2009/02/12. doi: 10.1097/JOM.0b013e318192bd2c . 19209041 10.1097/JOM.0b013e318192bd2c

[26] Nordin S, Palmquist E, Nordin M. Psychometric evaluation and normative data for a Swedish version of the Patient Health Questionnaire 15-Item Somatic Symptom Severity Scale. Scand J Psychol. 2013;54(2):112–7. Epub 2013/01/09. doi: 10.1111/sjop.12029 . 23294182 10.1111/sjop.12029

[27] National, Sleep, Foundation. What is Insomnia? [cited 2017 June 30th]. Available from: https://sleepfoundation.org/insomnia/content/what-is-insomnia.

[28] Von Korff M, Dworkin SF, Le Resche L. Graded chronic pain status: an epidemiologic evaluation. Pain. 1990;40(3):279–91. Epub 1990/03/01. . 2326094 10.1016/0304-3959(90)91125-3

[29] Bergstrom C, Hagberg J, Bodin L, Jensen I, Bergstrom G. Using a psychosocial subgroup assignment to predict sickness absence in a working population with neck and back pain. BMC Musculoskelet Disord. 2011;12:81 Epub 2011/04/28. doi: 10.1186/1471-2474-12-81 ; PubMed Central PMCID: PMC3097013. 21521502 10.1186/1471-2474-12-81 PMC3097013

[30] Ekblom-Bak E, Engström L-M, Ekblom Ö, Ekblom B. LIV 2000 Motionsvanor, fysisk prestationsförmåga och levnadsvanor bland svenska kvinnor och män i åldrarna 20–65 år LIV 2000 Exercise habits, physical ability and life habits among women and men in the ages 20–65 years. Stockholm: 2011.

[31] Sullivan M, Karlsson J, Ware JE Jr., The Swedish SF-36 Health Survey—I. Evaluation of data quality, scaling assumptions, reliability and construct validity across general populations in Sweden. Social science & medicine (1982). 1995;41(10):1349–58. . 8560302 10.1016/0277-9536(95)00125-q

[32] Alfoldi P, Dragioti E, Wiklund T, Gerdle B. Spreading of pain and insomnia in patients with chronic pain: results from a national quality registry (SQRP). J Rehabil Med. 2017;49(1):63–70. doi: 10.2340/16501977-2162 . 27904908 10.2340/16501977-2162

[33] Rubio-Arias JA, Marin-Cascales E, Ramos-Campo DJ, Hernandez AV, Perez-Lopez FR. Effect of exercise on sleep quality and insomnia in middle-aged women: A systematic review and meta-analysis of randomized controlled trials. Maturitas. 2017;100:49–56. doi: 10.1016/j.maturitas.2017.04.003 . 28539176 10.1016/j.maturitas.2017.04.003

[34] Tang NK, Lereya ST, Boulton H, Miller MA, Wolke D, Cappuccio FP. Nonpharmacological Treatments of Insomnia for Long-Term Painful Conditions: A Systematic Review and Meta-analysis of Patient-Reported Outcomes in Randomized Controlled Trials. Sleep. 2015;38(11):1751–64. doi: 10.5665/sleep.5158 ; PubMed Central PMCID: PMCPMC4813361. 25902806 10.5665/sleep.5158 PMC4813361

[35] World, Health, Organisation. WHO guidelines on basic training and safety in chiropractic 2005. http://apps.who.int/medicinedocs/documents/s14076e/se.pdf].

[36] Troiano RP, Berrigan D, Dodd KW, Masse LC, Tilert T, McDowell M. Physical activity in the United States measured by accelerometer. Med Sci Sports Exerc. 2008;40(1):181–8. Epub 2007/12/20. doi: 10.1249/mss.0b013e31815a51b3 . 18091006 10.1249/mss.0b013e31815a51b3

[37] Kanagasabai T, Thakkar NA, Kuk JL, Churilla JR, Ardern CI. Differences in physical activity domains, guideline adherence, and weight history between metabolically healthy and metabolically abnormal obese adults: a cross-sectional study. Int J Behav Nutr Phys Act. 2015;12:64 Epub 2015/05/20. doi: 10.1186/s12966-015-0227-z ; PubMed Central PMCID: PMC4490726. 25982079 10.1186/s12966-015-0227-z PMC4490726

